# Recent advances in immunotherapies: from infection and autoimmunity, to cancer, and back again

**DOI:** 10.1186/s13073-018-0588-4

**Published:** 2018-10-31

**Authors:** Samantha L. Bucktrout, Jeffrey A. Bluestone, Fred Ramsdell

**Affiliations:** 1 Parker Institute of Cancer Immunotherapy, 1 Letterman Drive, San Francisco, CA USA; 2 Diabetes Center, University of California, San Francisco, San Francisco, CA 94129 USA

## Abstract

For at least 300 years the immune system has been targeted to improve human health. Decades of work advancing immunotherapies against infection and autoimmunity paved the way for the current explosion in cancer immunotherapies. Pathways targeted for therapeutic intervention in autoimmune diseases can be modulated in the opposite sense in malignancy and infectious disease. We discuss the basic principles of the immune response, how these are co-opted in chronic infection and malignancy, and how these can be harnessed to treat disease. T cells are at the center of immunotherapy. We consider the complexity of T cell functional subsets, differentiation states, and extrinsic and intrinsic influences in the design, success, and lessons from immunotherapies. The integral role of checkpoints in the immune response is highlighted by the rapid advances in FDA approvals and the use of therapeutics that target the CTLA-4 and PD-1/PD-L1 pathways. We discuss the distinct and overlapping mechanisms of CTLA-4 and PD-1 and how these can be translated to combination immunotherapy treatments. Finally, we discuss how the successes and challenges in cancer immunotherapies, such as the collateral damage of immune-related adverse events following checkpoint inhibition, are informing treatment of autoimmunity, infection, and malignancy.

## Background

Modulation of the immune system to treat disease dates back to before the eighteenth century when the practice of inoculation with smallpox was used in India, China, and Africa before being adopted in Europe [1]. At the end of the nineteenth century William B. Coley injected a soft tissue sarcoma patient with streptococcal cultures. Following an acute attack of erysipelas, the tumor underwent extensive necrosis and the patient remained tumor free for 8 years [2]. Over time, Coley’s toxins were sidelined for emerging chemotherapy and radiation. While Coley hypothesized that the noxious nature of the bacterial products was directly causing the destruction of the tumor, our current understanding would suggest that Coley’s toxins initiated an immune response that attacked the tumor. Many of today’s cancer immunotherapy drugs are based on this principle. Thus, we have now come full circle and recognize that the principles that control the immune response to infection are also manifest in many normal physiological processes, in autoimmunity, and can also be harnessed to treat cancer.

## The T cell immune response in context

The immune response, whether to infection, in autoimmunity, or to cancer, is orchestrated by a multitude of distinct and specific cells. Interactions between dendritic cells and T cells are the primary pathway to generating immunity or tolerance [3]. However, T cells remain central, potent effectors of the response. T cell responses are characterized by vignettes of dynamic changes in CD4:CD8 T cell ratios, T effector (Teff) to regulatory T cell (Treg) ratios, and canonical T cell differentiation states such as naïve T, Teff, helper T cell subsets including Th1, Th2, Th17, central memory T (Tcm), tissue-resident memory cells (TRM), and exhausted T cells (Tex). Differentiation states are characterized by discrete epigenetic and transcriptional profiles, dynamic expression of molecules with functional consequences, metabolic changes, and differences in persistence [4–6]. Prolonged viral infection or high tumor burden with chronic T cell stimulation in challenging tissue environments, such as low oxygen, limited nutrients, or altered pH, results in terminal T cell exhaustion or unresponsiveness [7, 8]. The balance between factors such as reduced or reprogrammed Tex to Teff ratios have been associated with successful outcomes following cancer immunotherapy, antiviral therapy, or vaccination response, but with poor prognosis for autoimmunity [9, 10]. Indeed, it is the amalgam of many cellular interactions that both drive an immune response as well as determine the effectiveness for any given outcome.

## T cell immunotherapies

**Fig. 1:**
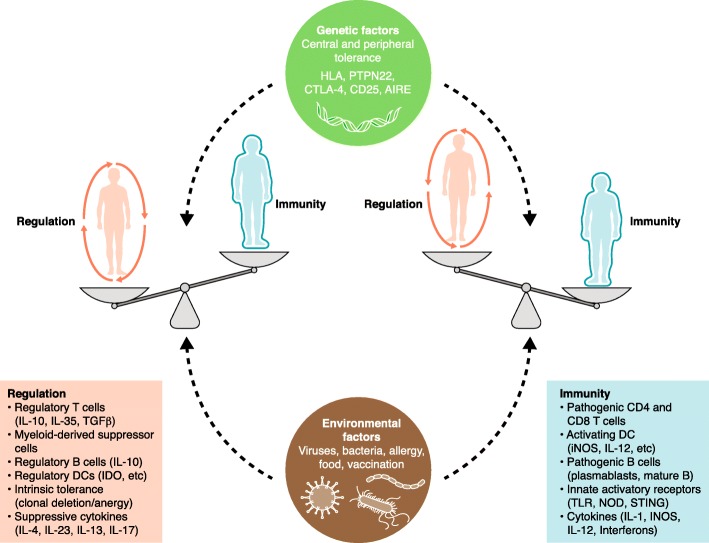
Immune health is a delicate balance between tolerance and immunity. *DC* dendritic cell, *iNOS* induced nitric oxide synthase

Our fundamental understanding of immunity has been fueled by tremendous technological advances in recent decades: the cloning of the human and mouse genomes, efficient and controlled editing of the mouse genome, high dimensional imaging, and the detailed analyses of both transcriptional and proteomic cellular properties (including at the single cell level). Following on from basic mechanistic studies, drugs targeting specific immune factors have proven to be effective in autoimmunity and additional pathways are under evaluation. Fast-track approvals of immunotherapies in a range of human malignancies are contributing to an explosion of preclinical and clinical research of the human immune system. What is emerging is that peripheral tolerance mechanisms that fail in autoimmunity are co-opted in progressive malignancies and chronic infections. Thus, pathways targeted for therapeutic intervention in autoimmune diseases can be modulated in the opposite sense in malignancy and infectious disease (Fig. 1).

The majority of clinically approved cancer immunotherapies have T cells central to their mechanism and fall broadly into two categories: (1) agents that directly target and modulate endogenous T cell responses; and (2) cellular therapies where modified T cells are the therapy. For the former, there are two general approaches: blockade of checkpoint molecule activity on T cells, which are currently the most powerful class of anticancer immunotherapies (discussed below), and agents that modulate the level of various cytokines that influence T cell behavior. One example of the latter is interleukin-2 (IL-2), a central growth factor for T cells and natural killer (NK) cells. IL-2 is produced by activated T cells and acts locally via a heterodimeric receptor comprising a high affinity α receptor (CD25), lower affinity β receptor (CD122), and a γ receptor (CD132) that signals cell survival, proliferation, and activation. CD4^+^ Foxp3^+^ Tregs constitutively express relatively high levels of CD25 and thus outcompete effector/memory CD4^+^ and CD8^+^ T cells and NK cells for limiting IL-2 [11]. Low doses of exogenous IL-2 give Tregs a competitive advantage and increase Treg:Teff ratios, having beneficial effects in preclinical models of multiple sclerosis, autoimmune diabetes, systemic lupus erythematosus (SLE), and graft versus host disease (GvHD) [12]. Low dose (LD) IL-2 is currently being evaluated in GvHD and SLE. A large bolus of IL-2 activates and expands NK cells and CD4^+^ and CD8^+^ T effector cells. High dose (HD) IL-2 was approved for metastatic renal cell carcinoma in 1992, and metastatic melanoma in 1998, improving survival in up to 8% and 17% of patients, respectively [13, 14]. However, the broad use of HD IL-2 has been limited because of toxicities of vascular leak syndrome and hypotension which may involve active IL-2Rs on endothelia [15]. Other γ-chain family cytokines have more favorable safety profiles, with similar immune T cell effects, and are under active investigation for therapeutic targeting. A deeper understanding and leverage of the subtle differences in cytokine:receptor binding, receptor usage and expression, and signaling pathways are giving rise to promising advances in targeting cytokines in both cancer and autoimmunity, such as recent work by Garcia, Bluestone, and colleagues [16] who engineered a synthetic IL-2R–IL-2 pair that effectively boosted engineered CD4^+^ and CD8^+^ T cell expansion in vivo and in vitro while limiting off-target effects and toxicity.

Advances in cellular therapies are being leveraged to promote immune suppression or cytotoxicity, for autoimmunity, cancer, and infectious disease. Examples include the expansion of autologous cells ex vivo for autoimmune diseases using Tregs, in malignancies with tumor infiltrating T cells (TILs), or in viral infection with CD8^+^ cytotoxic lymphocytes or NK cells, with some limited success. After initial disappointing trials in B cell malignancies, dramatic responses have led to recent Food and Drug Administration (FDA) approvals for autologous T cell therapies expressing CD19-targeted chimeric antigen receptors (CART) with co-stimulatory signaling domains [17, 18]. Approximately 50% of patients enrolled have successful T cell infusions, with 83% of the infused patients having clinical benefit [17]. Challenges for CART therapies include tumor escape by downregulation of the CAR target (CD19 escape variant loss), lack of CART persistence, and toxicities of least three discrete mechanisms that can be fatal [19, 20]. CARs are composed of single-chain fragments of monoclonal antibodies, which have considerably higher affinity than natural T cell receptors (TCRs), which may, in part, be the basis for the undesirable off-target effects of CART. Notwithstanding, the high success rate has created extraordinary interest in CART therapies in cancer with over 200 ongoing CART trials [19]. In order to address various mechanisms of resistance, many of these trials include advances in treatment regimens, combinations with other approved agents, and genetic modifications of the cells, including the use of CRISPR gene editing technologies. To date, T cell therapies have had limited success in solid tumors, which is an area of intense investigation. Multiple barriers to T cell trafficking and activity are presented by solid tumor microenvironments, including chronic antigen stimulation and lack of co-stimulatory checkpoints leading to exhaustion, limited nutrients and toxic metabolites, non-permissive stromal elements, and immune suppression [21]. Open questions remain, such as whether tolerance pathways will dominate over tumor rejection, whether transferred cells can be maintained long term, the extent of antigen loss, and the most effective approaches to address the suppressive tumor microenvironment of solid tumors. Further, it is unclear whether successful long-term responses will require engagement of the endogenous immune system. Many of these issues are reflective of the normal processes in generating an immune response to pathogens as well as the regulatory processes that limit immune-mediated damage to normal tissue. Lessons from cellular therapy approaches in cancer are providing advances in autoimmunity treatments, such as by cytotoxic targeting of pathogenic B cells [22], and arming Tregs with high-affinity TCRs for tissue-specific protein antigens [23].

## The integral role of checkpoints in the immune response

Fundamental murine and in vitro experiments as well as clinical experience have demonstrated that effector T cells are curtailed by multiple extrinsic and intrinsic factors, including: dependence on essential growth factors such as IL-2 that are limiting; downregulation of co-stimulatory molecules such as TNFRsf members and CD28; and increased expression of co-inhibitory receptors that function at discrete checkpoints to regulate homeostasis of the adaptive immune response, by dampening immune cell activation and/or effector functions. One such checkpoint, the co-inhibitory molecule CTLA-4, is absolutely required for post-thymic T cell tolerance and immune homeostasis [24]. Its absence by genetic deletion in mice or haploinsufficiency in patients results in heightened expression of co-stimulatory ligands by dendritic cells, rampant T cell expansion and activation, and autoimmunity [25, 26]. CTLA-4 attenuates T cell activation by regulating the CD28 co-stimulatory signals that are required for optimal activation. CTLA-4 competitively binds the co-stimulatory ligands CD80 and CD86 and can thus control T cell activation in instances where access to T cell co-stimulatory molecules is limited. Moreover, CTLA-4 actively removes CD80 and CD86 from dendritic cells [27], further limiting co-stimulation.

As the field has expanded, many other T cell inhibitory molecules have been described, such as PD-1, Tim-3, LAG-3, and TIGIT [28]. These targets are expressed coordinately in circumstances of immune tolerance, chronic infection, and inflammation and have both overlapping and distinct roles regulating immune responses (Table 1), and can, in some instances, compensate for the loss of CTLA-4 checkpoint interactions. The factors and mechanisms that influence expression and regulation of immune checkpoint molecules remain areas of intense investigation [29], though it is established that while most co-stimulatory molecules are downregulated and co-inhibitory molecules are upregulated following activation via TCR/CD28, in situations of chronic activation, such as in T cell infiltrated tumors or chronic infections, T cells express multiple co-inhibitory molecules [30, 31]. Moreover, Tregs constitutively express multiple co-inhibitory molecules that contribute to their stability and function [32], whose expression may be driven by the tonic TCR signaling Tregs experience in homeostasis. In other instances, checkpoint molecules engage distinct regulatory pathways either on activated T cells or on other cells mediating immunity. Additionally, the ligands for these receptors may be expressed in distinct locations, such as non-lymphoid tissues. Among the most well studied of these alternative checkpoints is PD-1, first discovered in 1992 by Honjo and colleagues [33]. This T cell checkpoint pathway (mediated through binding of ligands PD-L1 and PD-L2) was described to dampen responses of Teffs involved in antiviral immunity [34]. PD-1 deficiency results in accelerated and more severe autoimmune diseases and accelerated allograft rejection. In contrast, PD-1 ligand expression curtails T cell activation during acute infection and inflammation, protecting the heart, pancreas, and lung from immunopathology [35–37]. Upon binding PD-L1 or PD-L2, PD-1 directly attenuates TCR/CD28 signaling via the recruitment of tyrosine phosphatases to the immunoglobulin receptor switch motif and inhibitory motifs contained within the intracellular chain [38–40]. PD-1 and CTLA-4 have overlapping and discrete mechanisms of T cell regulation, and PD-1 abrogates TCR signals by dephosphorylating key signaling intermediates, including PI3K, Akt, Zap70, and PKCθ [41, 42]. The distinct mechanisms of action as well as distinct expression of ligands suggest that these molecules may function at different points in T cell activation. Consistent with this, deficiency in PD-1 and CTLA-4 can promote spontaneous autoimmunity even on genetic backgrounds that do not usually develop autoimmune disease [35, 43, 44], although the pattern and severity do not fully overlap. PD-1 and CTLA-4 have distinct spatial and temporal patterns of expression: CTLA-4 is rapidly mobilized at the surface during the early phases of antigen-mediated activation, whereas PD-1 is expressed during later differentiation stages, on memory effector cells within the CD8^+^ and CD4^+^, and Treg lineages, and increases with continued antigen expression [32, 45]. PD-1 expression on the cell surface is very stable, whereas surface CTLA-4 is rapidly removed by internalization. These distinct mechanisms are reflected by the results of combination therapy with CTLA-4 and PD-1/PD-L1 blocking antibodies which show synergy compared with either monotherapy for the treatment of metastatic melanoma [46]. Indeed, studies in mouse tumor models show that antiCTLA-4 expands effector CD4^+^ T cells and anti-PD-1 antibody “reinvigorates” exhausted-like CD8^+^ T cells within the tumor microenvironment [47]. The discovery that CTLA-4 initiated T cell anergy and PD-1-mediated T cell exhaustion has reframed our understanding of immunity and brought in an era of immune control in infectious diseases, autoimmunity, and cancer immunology.

**Table 1:** Immune checkpoint molecules being targeted by therapeutics for cancer, infectious disease, or autoimmunity

Checkpoint molecule	Biological role	Therapeutic	Disease	Reference
CTLA-4	Inhibits TCR/CD28 signaling.	Ipilimumab	Malignancy	Schadendorf et al. 2015 [93]
	Limits primary T cell activation.	Tremelimumab	Malignancy	Ribas et al. 2013 [94]
			Chronic infection	Sangro et al. 2013 [95]
		Abatacept	Autoimmune disease	Kremer et al. 2006 [96]
PD-1/PD-L1	Inhibits TCR/CD28 -signaling via ITIM and ITSM. Limits T e ffector function.	Nivolumab	Malignancy	Topalian et al. 2012 [49]
		Pembrolizumab	Malignancy	Reck et al. 2016 [97]
		Avelumab	Malignancy	Kaufman et al. 2016 [98]
		Atezolizumab	Malignancy	Rittmeyer et al. 2017 [99]
TIGIT	Inhibits CD226 co-stimulation via ITIM. Limits T cell e ffector function.	OMP-31 M32	Malignancy	NCT03 119,428
		MTIG7192 A	Malignancy	NCT03563716
		BMS-986207	Malignancy	NCT02913313
Tim3 (HAVCR2)	Negatively regulates TCR/CD28 signaling. Limits T cell activation.	Ly3321367	Malignancy	NCT03099109
		MBG453	Malignancy	NCT02608268
		TSR-022	Malignancy	NCT02817633
		Sym023	Malignancy	NCT03489343
LAG-3	Negatively regulates TCR signaling. Limits T cell proliferation.	BMS-986016	Malignancy	NCT01968109
		TSR-033	Malignancy	NCT03250832
		MGD013	Malignancy	NCT03219268
		Sym022	Malignancy	NCT03489369
		IMP321	Malignancy	NCT02676869
		GSK2831781	Autoimmunity	NCT02195349

## Immunological mechanisms: Lessons from the clinic

Remarkably durable responses in subsets of cancer patients receiving CTLA-4 and PD-1/PD-L1 antibodies have driven fast-track approvals by the FDA for a range of malignancies, where long-lasting, extended survival times range from 24 to 45% [48, 49]. Antibodies that block CTLA-4 extrinsic and intrinsic immune regulation (ipilimumab, tremilimumab) result in clinical responses that correlate with the emergence of new high-avidity T cell clones and anti-tumor T cell clones [50, 51], suggesting that the site of action is in lymphoid tissue. Another mechanism of action of the therapeutic drug is provided by the drug design. For example, ipilimumab is a humanized IgG1 recombinant antibody that can mediate antibody-dependent cellular cytotoxicity (ADCC) and complement-mediated cellular cytotoxicity, and may deplete tumor-infiltrating Tregs, which have elevated expression of CTLA-4 compared with Teff cells [52, 53]. It is tempting to compare the clinical experience of ipilimumab with that of tremilimumab, a hIgG4 anti-CTLA-4 that is less functional for ADCC than ipilimumab, to gain insights into the role of tumor Tregs, FcγR-expressing NK cells and macrophages in the antitumor response, and immune-related adverse events (see below). Thus, by design, immune therapeutics can provide more complex information that can illuminate previously unexplored biology. In autoimmunity, recombinant CTLA-4Ig (abatacept) dampens the immune response by blocking the co-stimulatory ligands CD80 and CD86 [54], thus regulating the extent of CD28 co-stimulation, and abatacept is approved for subtypes of arthritis. Currently five PD-1/PD-L1 targeting antibodies are approved for cancer treatment (Table 1), with dozens more in development. At present, these are generally approved for advanced stages of metastatic melanoma, non-small cell lung cancer, non-Hodgkin lymphoma, head and neck cellular carcinoma, and any unresectable or metastatic solid tumor with microsatellite instability (MSI) or DNA mismatch repair deficiency (DMRD) [55]. Higher response rates to immune checkpoint inhibition are seen in tumors with high mutational burden, such as MSI-high or DMRD tumors [56–58]. Higher somatic mutational burden resulting in increased neoantigen generation is the putative mechanism for the increased response rates to checkpoint inhibition for these tumors [59–61]. Similarly, encouraging data are emerging of increased clinical efficacy with the combination of immune checkpoint inhibition and vaccination. Many cancer vaccines, be they dendritic cell or viral based or DNA/RNA expressing tumor-associated antigens, have produced modest or negative results [62–64], suggesting additional agents are needed. Indeed, combinations of various cancer vaccines with ipilimumab in the priming phase and nivolumab concurrent with or sequentially following vaccination have shown promising early signs of clinical benefit compared with control arms or historic datasets [48, 65–67]. The majority of the data collected in the clinic support that PD-1/PD-L1 blockade works because of a pre-existing anti-tumor CD8^+^ T cell response. There appears to be nothing in the drug design that distinguishes the anti-PD-1 antibodies, but the anti-PD-L1 antibodies, similar to CTLA-4, are either hIgG1 (avelumab) or hIgG4 (atezolizumab). Both avelumab and atezolizumab have followed approvals of anti-PD-1 antibodies in indications that were not fully investigated with other checkpoint inhibitors, including urothelial cancers and Merkel cell carcinoma, respectively, with similar response profiles. Further data sets and deep translational analysis of the responses will be required to elucidate the role of cell depletion versus blocking in the clinical and immune response of targeting PD-L1 pathways. The successful activation of CD8 T cells by blocking PD-1/PD-L1 also suggests that a drug that actively triggers this pathway could be useful in autoimmunity. No such drugs are in clinical trials at this point, potentially highlighting challenges in protein engineering or reliable agonism in vivo.

The clinical experience with immunotherapy has already provided valuable lessons about fundamental immune mechanisms, including the role of the tumor microenvironment (TME), alternative checkpoint pathways, and the relevant roles of different checkpoints at different stages and locations of disease. However, there is a fine line between engaging the immune response to eradicate tumors and preventing collateral damage from self- and cross-reactive T cells and boosted inflammation [20]. A majority of patients receiving immune checkpoint inhibition (ICI) experience an immune-related adverse event (irAE). A grade 3–4 irAE requires intervention and, in most cases, cessation of immunotherapy, which accounts for 13% of patients treated with anti-PD-1, 23% treated with anti CTLA-4, and 55% of those treated with the combination of PD-1 and CTLA-4 blockade [48, 68, 69]. irAEs can have manifestations in nearly every tissue and organ, the most common being in barrier tissues such as skin and gastrointestinal and respiratory organs, whereas those in internal organs like heart, central nervous system, and pancreas are rare [70, 71]. Interestingly, the tissue(s) affected by the irAE correlates with the molecular target of the checkpoint therapy, rather than the organ of origin of the tumor, suggesting either that checkpoints of peripheral tolerance are tissue biased or that tissue-specific inflammation and/or pre-existing conditions affect the incidence and severity of the irAEs. For instance, the gut is highly susceptible to irAEs after anti-CTLA-4 therapy. In mice, modulating CTLA-4 signaling in Tregs suggests that blockade of intrinsic negative signaling is not the mechanism of colitis [72]. The mechanisms of action of ipilimumab is being tested in the clinic with smart drug design. New generation CTLA-4 antibodies designed on preclinical data are currently in clinical trials with the goal of reducing systemic immune-related toxicity while maintaining efficacy. The first is a conditionally active CTLA-4 antibody whose CDR3 regions that bind antigen are masked with a polypeptide attached to the framework region with a protease-cleaveable linker, resulting in a higher concentration of active CTLA-4 antibodies at the tumor site due to heightened protease activity [73]. In the second case, the Fc region has been engineered to have a higher affinity for activating FcγR, thus decreasing the threshold for antibody-dependent cellular cytotoxicity testing pre-clinical data indicating that biased depletion of tumor-associated Treg is important for efficacy [52]. In both of these cases, the goal is to be able to find a pharmacological method to achieve CTLA-4-based activity directed towards the tumor and sparing normal tissues. These phase I studies will potentially distinguish cell intrinsic mechanisms versus Tregs in the function of CTLA-4 in peripheral tolerance, particularly the gut, and the spatial, temporal characteristics of the CTLA-4:CD28 pathway in humans.

It is important to reiterate that a majority of patients undergoing immunotherapy treatment experience an irAE. These side effects are sometimes inflammatory in nature and can be reversed by short-term steroid treatment. In other instances, the irAEs are more severe and express characteristics of an autoimmune syndrome where steroids can abrogate severity but not always reverse the toxicity induced by the treatment. Many questions remain about the nature of this collateral damage, whether the patient had a pre-existing condition or whether the drugs, especially the newer combinations, are impacting incidence and severity. Mechanistic studies are underway to understand the relationship between the irAE and the anti-tumor response to ensure interventions to control the irAE do not blunt the anti-tumor response. The mechanisms central to both anti-tumor/anti-pathogen and autoimmunity are highly complementary, broadly including factors such as host genetics, environmental stimuli, prior exposure, and epigenetic status (Fig. 1). Thus, uncoupling irAE from anti-tumor response may rely on smart drug design for more precise delivery, such as masked antibodies that can be activated by proteases that are enriched in the tumor microenvironment, and intervention, including timing and duration of various interventions. Recent studies show that patients with pre-existing autoimmune diseases treated with ICI have a better chance of response across tumor indications than patients with no evidence of autoimmunity [74, 75]. Moreover, patients that experience an irAE following ICI treatment have better overall outcome if the irAE is managed. Generally, the limited use of steroids that manages the irAE appears not to restrict the immune response to the tumor [48, 76, 77], implying there may be “windows of opportunity” or dosing strategies that separate these functional outcomes. Genetics is likely to be another important factor in both tumor response and irAEs. For example, HLA has the largest influence on susceptibility to autoimmune disease, and heterozygosity within the MHC I HLA loci (A, B, and C) is associated with improved outcome for cancer patients treated with checkpoint inhibitors [78]. Once these many factors are better understood, risk evaluations for an irAE may become part of the decision criteria for immunotherapy selection and targeted interventions can be explored. As important, the study of irAEs may provide unique insights into the basis of autoimmunity and pathways targeted with this new class of cancer drugs may be repositioned for autoimmune disease interventions. Unlike chemotherapy or radiation therapy, immuno-oncology is based on the ability to either release or generate an effective immune response (in this case to a tumor). Decades of data have demonstrated that this is an organized process with dozens of specific pathways that need to be engaged in a particular order. Careful mechanistic studies of immunotherapy clinical studies have and will shed important light on how these pathways operate in humans during disease.

There are other implications of ICI outside cancer. Will it be possible to alter these regulatory pathways to develop therapies that can be exploited in infectious diseases and autoimmunity? Will the same targets, PD-1, CTLA-4, etc., play distinct roles in the infectious disease setting and can they be harnessed for vaccine development? Will these pathways be important for other aspects of an immune response that is not revealed by cancer biology (e.g., a potential role for PD-1 in generation of memory)? Will other co-inhibitory pathways be more or less important in such settings? Further clinical trials targeting some of these pathways (LAG3, Tim3, others) should provide insight into the roles of these pathways in the context of a human immune response. Finally, will the advent of the field of cancer immunotherapy mimic in some ways autoimmunity, where an immune response normally kept in check is unleashed to orchestrate immune-mediated tissue damage?

## Emerging complexities in checkpoint inhibition therapy

Thousands of oncology patients worldwide are now being treated with immunotherapy, driven by unprecedented examples of long-lasting responses in patients with metastases that are being described as cures. Successful cancer immunotherapy and vaccination generates immune memory for long-lasting protection. Despite remarkable progress, however, the majority of patients still do not respond to CTLA-4 or PD-1/PD-L1 blockade [79]. To understand sensitivity and resistance to immune checkpoint inhibition therapy, there has been a focus on aspects of tumor intrinsic properties and the host immune system. Tumors that have an immune infiltrate with a high proportion of CD8^+^ T cells and/or interferon (IFN) signature (sometimes referred to as “hot” or “inflamed”) respond better than those with a macrophage dominant or sparse immune infiltrate (sometimes referred to as “warm/cold” or “immune dessert”) [79–81]. Tumors with higher mutational burden tend to have more immune infiltrates, but may have increased propensity for immunoediting, for example, dysregulation of genes that are checkpoints in MHC-peptide presentation, avoiding recognition by T cells and activation of the WNT pathway that associates with reduced immune infiltrate and reduced sensitivity to immune checkpoint inhibitory therapies [82, 83]. Moreover, immune infiltrate can be a “double-edged sword”, as products of effector immune responses, such as IFNγ, drive expression of immune checkpoint inhibitors in the tumor microenvironment, including PD-L1, IDO-1, etc. [84].

As investigators work to understand immune limitations, there has been a focus on the characterization of the intrinsic factors that control T cell activity. T cell exhaustion in cancer shares hallmarks of exhaustion in response to chronic infection, including lack of proliferative capacity, increased expression of co-inhibitory molecules (PD-1, CTLA-4, VISTA, Tim3, LAG-3, 2B4), downregulation of effector molecules like IL-2, IFNγ, and TNFα, and associated Teff cell lineage-determining transcription factors such as Tbet and eomes [85, 86]. Terminal T cell exhaustion has been implicated in lack of response to anti-PD-1 therapy [86], so improved understanding of early mechanisms of exhaustion is an area of intense investigation. For example, epigenetic landscapes associated with CD8^+^ T cell exhaustion are being interrogated, and identification of functional enhancers that regulate phenotype, such as PD-1 expression, may be therapeutic targets. T cell activation per se drives permissibility to exhaustion. TCR signaling results in the nuclear localization of the transcription factor NFAT, and multiple gene proximal and distal enhancer regions have been described as NFAT-binding sites for PD-1 expression [87]. Emerging data from cancer patients is unclear as to the prognostic value of markers of T cell exhaustion in predicting response in immunotherapy, where the relative frequency of PD-1hi T cells have been shown to negatively [21] or positively [88, 89] predict response to immune checkpoint inhibition. Whether the differences are due to the markers used, tumor indication, or simply to the low numbers of patients analyzed, an increased understanding will evolve as technologies become standardized and a consensus of data develops. Use of current technologies such as single-cell transcriptome profiling, epigenetic analyses, TCR repertoire analysis, proteomics, and high-dimensional imaging of the spatial and temporal activities of a multitude of cell types on patient samples before and on immunotherapy is and will continue to provide unique and exciting insights into the human immune response to disease states and therapeutic intervention like never before.

Layered onto T cell intrinsic inhibition of effective, long-lasting anti-tumor responses, the TME presents multiple barriers to immune activation and effector function. Tumor cell (or TME) expression of immune regulatory proteins and pathways, including PD-L1, TGF-β, IDO-1, and iNOS, high myeloid suppressor cell and Treg:Teff cell ratios, stroma that create a physical barrier to immune cell entry and limited nutrients, low oxygen, and low pH are associated with poor prognosis and resistance to checkpoint blockade immunotherapy [90, 91]. Understanding recent clinical failures (e.g., IDO-1 antagonists) and the lack of correlation between PD-L1 expression and response to anti-PD-L1 highlight the need to define where particular drugs principally act—within the tumor or in a lymphoid organ/organoid. For example, recent data point to the role of TGFβ in lymphocyte exclusion which suggests particular tumor subtypes and combinations that are relevant for anti-TGFβ therapeutics [92]. Therapies directed at priming a response might function at very different locations to those that target an effector response. Further, efforts to re-polarize/block the activity of the suppressor myeloid compartment and to recruit and engage cross-presenting dendritic cells are underway. Trials with various biologics, small molecules, and emerging technologies for direct tumor delivery (oncolytic viruses, nanoparticles, intra-tumoral injection, etc.) should generate key insights into the role of many pathways important for the generation of a successful response (cytokines, STING and TLR agonists, CD40, CCR2, CXCR2, PI3Kγ).

## Conclusions

Oncology is undergoing an unprecedented shift in thinking, integrating the tumor molecular profile, the microenvironment, and the immune profile to give a more holistic view of tumor–immune interactions that should drive future treatment decisions [90]. Mechanistic studies of irAEs reveal that distinct checkpoints are dominant for peripheral tolerance for certain tissues and organs, thus identifying targets for the natural autoimmune diseases of that organ. Studies on the activity of vaccines, the role of new checkpoint molecules, new pathways for stimulation of innate responses, and even the genetic determinants of response will all inform both basic immune mechanisms and have applications in the generation of effective immunity to pathogens. The implications of these principles are already being considered in the context of infectious disease (both vaccination and treatment) as well as what this can tell us about treatments for chronic autoimmunity. Decades of work on the principles of fundamental immunity are now bearing fruit in the treatment of cancer—and the study of the immunity of cancer is returning the favor.

## Abbreviations

ADCC: Antibody-dependent cellular cytotoxicity
CART: T cells with chimeric antigen receptor
DMRD: DNA mismatch repair deficiency
FDA: Food and Drug Administration
GvHD: Graft versus host disease
HD: High dose
ICI: Immune checkpoint inhibition
IL-2: Interleukin-2
irAE: Immune-related adverse event
LD: Low dose
MSI: Microsatellite instability
NK: Natural killer
Tcm: Central memory T cell
TCR: T cell receptor
Teff: Effector T cell
Tex: Exhausted T cell
TIL: Tumor infiltrating lymphocytes
TME: Tumor microenvironment
Treg: Regulatory T cell
TRM: Tissue-resident memory

## References

[1] Riedel S (2005). Edward Jenner and the history of smallpox and vaccination. Proc (Bayl Univ Med Cent)..

[2] Starnes CO (1992). Coley’s toxins in perspective. Nature.

[3] Steinman RM (2012). Decisions about dendritic cells: past, present, and future. Annu Rev Immunol.

[4] Buck MD, O’Sullivan D, Pearce EL (2015). T cell metabolism drives immunity. J Exp Med..

[5] O’Shea JJ, Paul WE (2010). Mechanisms underlying lineage commitment and plasticity of helper CD4+ T cells. Science.

[6] Pauken KE, Sammons MA, Odorizzi PM, Manne S, Godec J, Khan O (2016). Epigenetic stability of exhausted T cells limits durability of reinvigoration by PD-1 blockade. Science.

[7] Sen DR, Kaminski J, Barnitz RA, Kurachi M, Gerdemann U, Yates KB (2016). The epigenetic landscape of T cell exhaustion. Science.

[8] Philip M, Fairchild L, Sun L, Horste EL, Camara S, Shakiba M (2017). Chromatin states define tumour-specific T cell dysfunction and reprogramming. Nature.

[9] Huang AC, Postow MA, Orlowski RJ, Mick R, Bengsch B, Manne S (2017). T-cell invigoration to tumour burden ratio associated with anti-PD-1 response. Nature.

[10] EF MK, Lee JC, DRW J, Lyons PA, KGC S (2015). T-cell exhaustion, co-stimulation and clinical outcome in autoimmunity and infection. Nature.

[11] O’Gorman WE, Dooms H, Thorne SH, Kuswanto WF, Simonds EF, Krutzik PO (2009). The initial phase of an immune response functions to activate regulatory T cells. J Immunol.

[12] Klatzmann D, Abbas AK (2015). The promise of low-dose interleukin-2 therapy for autoimmune and inflammatory diseases. Nat Rev Immunol.

[13] Fisher RI, Rosenberg SA, Fyfe G (2000). Long-term survival update for high-dose recombinant interleukin-2 in patients with renal cell carcinoma. Cancer J Sci Am.

[14] Rosenberg SA, Yang JC, White DE, Steinberg SM (1998). Durability of complete responses in patients with metastatic cancer treated with high-dose interleukin-2: identification of the antigens mediating response. Ann Surg.

[15] Epstein AL, Mizokami MM, Li J, Hu P, Khawli LA (2003). Identification of a protein fragment of interleukin 2 responsible for vasopermeability. J Natl Cancer Inst.

[16] Sockolosky JT, Picton L, Su LL, Le AC, Chhabra A, Silveria SL (2018). Selective targeting of engineered T cells using orthogonal IL-2 cytokine-receptor complexes. Science.

[17] Park JH, Rivière I, Gonen M, Wang X, Sénéchal B, Curran KJ (2018). Long-term follow-up of CD19 CAR therapy in acute lymphoblastic leukemia. N Engl J Med.

[18] Lee DW, Kochenderfer JN, Stetler-Stevenson M, Cui YK, Delbrook C, Feldman SA (2015). T cells expressing CD19 chimeric antigen receptors for acute lymphoblastic leukaemia in children and young adults: a phase 1 dose-escalation trial. Lancet.

[19] June CH, O’Connor RS, Kawalekar OU, Ghassemi S, Milone MC (2018). CAR T cell immunotherapy for human cancer. Science.

[20] June CH, Warshauer JT, Bluestone JA (2017). Is autoimmunity the Achilles’ heel of cancer immunotherapy. Nat Med.

[21] Anderson KG, Stromnes IM, Greenberg PD (2017). Obstacles posed by the tumor microenvironment to T cell activity: a case for synergistic therapies. Cancer Cell.

[22] Ellebrecht CT, Bhoj VG, Nace A, Choi EJ, Mao X, Cho MJ (2016). Reengineering chimeric antigen receptor T cells for targeted therapy of autoimmune disease. Science.

[23] Yeh WI, Seay HR, Newby B, Posgai A, Moniz FB, Michels A (2017). Avidity and bystander suppressive capacity of human regulatory T cells expressing de novo autoreactive T-cell receptors in type 1 diabetes. Front Immunol.

[24] Walunas TL, Lenschow DJ, Bakker CY, Linsley PS, Freeman GJ, Green JM (1994). CTLA-4 can function as a negative regulator of T cell activation. Immunity.

[25] Waterhouse P, Penninger JM, Timms E, Wakeham A, Shahinian A, Lee KP (1995). Lymphoproliferative disorders with early lethality in mice deficient in Ctla-4. Science.

[26] Kuehn HS, Ouyang W, Lo B, Deenick EK, Niemela JE, Avery DT (2014). Immune dysregulation in human subjects with heterozygous germline mutations in CTLA4. Science.

[27] Qureshi OS, Zheng Y, Nakamura K, Attridge K, Manzotti C, Schmidt EM (2011). Trans-endocytosis of CD80 and CD86: a molecular basis for the cell-extrinsic function of CTLA-4. Science.

[28] Mahoney KM, Rennert PD, Freeman GJ (2015). Combination cancer immunotherapy and new immunomodulatory targets. Nat Rev Drug Discov.

[29] Hui E, Cheung J, Zhu J, Su X, Taylor MJ, Wallweber HA (2017). T cell costimulatory receptor CD28 is a primary target for PD-1-mediated inhibition. Science.

[30] Hung AL, Maxwell R, Theodros D, Belcaid Z, Mathios D, Luksik AS (2018). TIGIT and PD-1 dual checkpoint blockade enhances antitumor immunity and survival in GBM. Oncoimmunology.

[31] Harris-Bookman S, Mathios D, Martin AM, Xia Y, Kim E, Xu H, et al. Expression of LAG-3 and efficacy of combination treatment with anti-LAG-3 and anti-PD-1 monoclonal antibodies in glioblastoma. Int J Cancer. 2018. 10.1002/ijc.31661. 10.1002/ijc.31661 PMC7105259 30248181

[32] Wing K, Onishi Y, Prieto-Martin P, Yamaguchi T, Miyara M, Fehervari Z (2008). CTLA-4 control over Foxp3+ regulatory T cell function. Science.

[33] Ishida Y, Agata Y, Shibahara K, Honjo T (1992). Induced expression of PD-1, a novel member of the immunoglobulin gene superfamily, upon programmed cell death. EMBO J.

[34] Iwai Y, Terawaki S, Ikegawa M, Okazaki T, Honjo T (2003). PD-1 inhibits antiviral immunity at the effector phase in the liver. J Exp Med.

[35] Nishimura H, Okazaki T, Tanaka Y, Nakatani K, Hara M, Matsumori A (2001). Autoimmune dilated cardiomyopathy in PD-1 receptor-deficient mice. Science.

[36] Fife BT, Guleria I, Gubbels Bupp M, Eagar TN, Tang Q (2006). Insulin-induced remission in new-onset NOD mice is maintained by the PD-1-PD-L1 pathway. J Exp Med.

[37] Lucas JA, Menke J, Rabacal WA, Schoen FJ, Sharpe AH, Kelley VR (2008). Programmed death ligand 1 regulates a critical checkpoint for autoimmune myocarditis and pneumonitis in MRL mice. J Immunol.

[38] Chemnitz JM, Parry RV, Nichols KE, June CH, Riley JL (2004). SHP-1 and SHP-2 associate with immunoreceptor tyrosine-based switch motif of programmed death 1 upon primary human T cell stimulation, but only receptor ligation prevents T cell activation. J Immunol.

[39] Freeman GJ, Long AJ, Iwai Y, Bourque K, Chernova T, Nishimura H (2000). Engagement of the PD-1 immunoinhibitory receptor by a novel B7 family member leads to negative regulation of lymphocyte activation. J Exp Med.

[40] Latchman Y, Wood CR, Chernova T, Chaudhary D, Borde M, Chernova I (2001). PD-L2 is a second ligand for PD-1 and inhibits T cell activation. Nat Immunol.

[41] Parry RV, Chemnitz JM, Frauwirth KA, Lanfranco AR, Braunstein I, Kobayashi SV (2005). CTLA-4 and PD-1 receptors inhibit T-cell activation by distinct mechanisms. Mol Cell Biol.

[42] Fife BT, Pauken KE, Eagar TN, Obu T, Wu J, Tang Q (2009). Interactions between PD-1 and PD-L1 promote tolerance by blocking the TCR-induced stop signal. Nat Immunol.

[43] Nishimura H, Nose M, Hiai H, Minato N, Honjo T (1999). Development of lupus-like autoimmune diseases by disruption of the PD-1 gene encoding an ITIM motif-carrying immunoreceptor. Immunity.

[44] Tivol EA, Borriello F, Schweitzer AN, Lynch WP, Bluestone JA, Sharpe AH (1995). Loss of CTLA-4 leads to massive lymphoproliferation and fatal multiorgan tissue destruction, revealing a critical negative regulatory role of CTLA-4. Immunity.

[45] Gros A, Robbins PF, Yao X, Li YF, Turcotte S, Tran E (2014). PD-1 identifies the patient-specific CD8^+^ tumor-reactive repertoire infiltrating human tumors. J Clin Invest.

[46] Wolchok JD, Kluger H, Callahan MK, Postow MA, Rizvi NA, Lesokhin AM (2013). Nivolumab plus ipilimumab in advanced melanoma. N Engl J Med.

[47] Wei SC, Levine JH, Cogdill AP, Zhao Y, NAS A, Andrews MC (2017). Distinct cellular mechanisms underlie anti-CTLA-4 and anti-PD-1 checkpoint blockade. Cell.

[48] Hodi FS, O'Day SJ, DF MD, Weber RW, Sosman JA, Haanen JB (2010). Improved survival with ipilimumab in patients with metastatic melanoma. New Engl J Med.

[49] Topalian SL, Hodi FS, Brahmer JR, Gettinger SN, Smith DC, DF MD (2012). Safety, activity, and immune correlates of anti–PD-1 antibody in cancer. New Engl J Med.

[50] Kvistborg P, Philips D, Kelderman S, Hageman L, Ottensmeier C, Joseph-Pietras D (2014). Anti-CTLA-4 therapy broadens the melanoma-reactive CD8+ T cell response. Sci Transl Med.

[51] Cha E, Klinger M, Hou Y, Cummings C, Ribas A, Faham M (2014). Improved survival with T cell clonotype stability after anti-CTLA-4 treatment in cancer patients. Sci Transl Med.

[52] Simpson TR, Li F, Montalvo-Ortiz W, Sepulveda MA, Bergerhoff K, Arce F (2013). Fc-dependent depletion of tumor-infiltrating regulatory T cells co-defines the efficacy of anti-CTLA-4 therapy against melanoma. J Exp Med.

[53] Romano E, Kusio-Kobialka M, Foukas PG, Baumgaertner P, Meyer C, Ballabeni P (2015). Ipilimumab-dependent cell-mediated cytotoxicity of regulatory T cells ex vivo by nonclassical monocytes in melanoma patients. Proc Natl Acad Sci U S A.

[54] Lenschow DJ, Zeng Y, Thistlethwaite JR, Montag A, Brady W, Gibson MG (1992). Long-term survival of xenogeneic pancreatic islet grafts induced by CTLA4lg. Science.

[55] Lemery S, Keegan P, Pazdur R (2017). First FDA approval agnostic of cancer site – when a biomarker defines the indication. N Engl J Med.

[56] Van Allen EM, Miao D, Schilling B, Shukla SA, Blank C, Zimmer L (2015). Genomic correlates of response to CTLA-4 blockade in metastatic melanoma. Science.

[57] Rizvi NA, Hellmann MD, Snyder A, Kvistborg P, Makarov V, Havel JJ (2015). Cancer immunology. Mutational landscape determines sensitivity to PD-1 blockade in non-small cell lung cancer. Science.

[58] Hellmann MD, Nathanson T, Rizvi H, Creelan BC, Sanchez-Vega F, Ahuja A (2018). Genomic features of response to combination immunotherapy in patients with advanced non-small-cell lung cancer. Cancer Cell.

[59] Diaz LA, Le DT (2015). PD-1 blockade in tumors with mismatch-repair deficiency. N Engl J Med.

[60] Yarchoan M, Johnson BA, Lutz ER, Laheru DA, Jaffee EM (2017). Targeting neoantigens to augment antitumour immunity. Nat Rev Cancer.

[61] Yarchoan M, Hopkins A, Jaffee EM (2017). Tumor mutational burden and response rate to PD-1 inhibition. N Engl J Med.

[62] Kantoff PW, Higano CS, Shore ND, Berger ER, Small EJ, Penson DF (2010). Sipuleucel-T immunotherapy for castration-resistant prostate cancer. N Engl J Med.

[63] Andtbacka RH, Ross M, Puzanov I, Milhem M, Collichio F, Delman KA (2016). Patterns of clinical response with talimogene laherparepvec (T-VEC) in patients with melanoma treated in the OPTiM phase III clinical trial. Ann Surg Oncol.

[64] Collins JM, Redman JM, Gulley JL (2018). Combining vaccines and immune checkpoint inhibitors to prime, expand, and facilitate effective tumor immunotherapy. Expert Rev Vaccines.

[65] Ribas A, Dummer R, Puzanov I, VanderWalde A, RHI A, Michielin O (2017). Oncolytic virotherapy promotes intratumoral T cell infiltration and improves anti-PD-1 immunotherapy. Cell.

[66] Weber JS, Kudchadkar RR, Yu B, Gallenstein D, Horak CE, Inzunza HD (2013). Safety, efficacy, and biomarkers of nivolumab with vaccine in ipilimumab-refractory or -naive melanoma. J Clin Oncol.

[67] Gibney GT, Kudchadkar RR, RC DC, Thebeau MS, Czupryn MP, Tetteh L (2015). Safety, correlative markers, and clinical results of adjuvant nivolumab in combination with vaccine in resected high-risk metastatic melanoma. Clin Cancer Res.

[68] Hamid O, Robert C, Daud A, Hodi FS, Hwu WJ, Kefford R (2013). Safety and tumor responses with lambrolizumab (anti-PD-1) in melanoma. N Engl J Med.

[69] Weber JS, Kähler KC, Hauschild A (2012). Management of immune-related adverse events and kinetics of response with ipilimumab. J Clin Oncol.

[70] Weber JS, Hodi FS, Wolchok JD, Topalian SL, Schadendorf D, Larkin J (2017). Safety profile of nivolumab monotherapy: a pooled analysis of patients with advanced melanoma. J Clin Oncol.

[71] Moslehi JJ, Salem JE, Sosman JA, Lebrun-Vignes B, Johnson DB (2018). Increased reporting of fatal immune checkpoint inhibitor-associated myocarditis. Lancet.

[72] Kong KF, Fu G, Zhang Y, Yokosuka T, Casas J, Canonigo-Balancio AJ (2014). Protein kinase C-η controls CTLA-4-mediated regulatory T cell function. Nat Immunol.

[73] Drag M, Salvesen GS (2010). Emerging principles in protease-based drug discovery. Nat Rev Drug Discov.

[74] Johnson DB, Sullivan RJ, Ott PA, Carlino MS, Khushalani NI, Ye F (2016). Ipilimumab therapy in patients with advanced melanoma and preexisting autoimmune disorders. JAMA Oncol.

[75] Menzies AM, Johnson DB, Ramanujam S, Atkinson VG, ANM W, Park JJ (2017). Anti-PD-1 therapy in patients with advanced melanoma and preexisting autoimmune disorders or major toxicity with ipilimumab. Ann Oncol.

[76] Weber JS, O'Day S, Urba W, Powderly J, Nichol G, Yellin M (2008). Phase I/II study of ipilimumab for patients with metastatic melanoma. J Clin Oncol.

[77] Freeman-Keller M, Kim Y, Cronin H, Richards A, Gibney G, Weber JS (2016). Nivolumab in resected and unresectable metastatic melanoma: characteristics of immune-related adverse events and association with outcomes. Clin Cancer Res.

[78] Chowell D, LGT M, Grigg CM, Weber JK, Samstein RM, Makarov V (2018). Patient HLA class I genotype influences cancer response to checkpoint blockade immunotherapy. Science.

[79] Sharma P, Allison JP (2015). The future of immune checkpoint therapy. Science.

[80] Chen DS, Mellman I (2017). Elements of cancer immunity and the cancer-immune set point. Nature.

[81] Taube JM, Galon J, Sholl LM, Rodig SJ, Cottrell TR, Giraldo NA (2018). Implications of the tumor immune microenvironment for staging and therapeutics. Mod Pathol.

[82] Grasso CS, Giannakis M, Wells DK, Hamada T, Mu XJ, Quist M (2018). Genetic mechanisms of immune evasion in colorectal cancer. Cancer Discov.

[83] Deniger DC, Pasetto A, Robbins PF, Gartner JJ, Prickett TD, Paria BC, et al. T-cell responses to TP53 “Hotspot” mutations and unique neoantigens expressed by human ovarian cancers. Clin Cancer Res. 2018. 10.1158/1078-0432.CCR-18-0573. 10.1158/1078-0432.CCR-18-0573 PMC6239943 29853601

[84] Gajewski TF, Corrales L, Williams J, Horton B, Sivan A, Spranger S (2017). Cancer immunotherapy targets based on understanding the T cell-inflamed versus non-T cell-inflamed tumor microenvironment. Adv Exp Med Biol.

[85] Fridman WH, Pagès F, Sautès-Fridman C, Galon J (2012). The immune contexture in human tumours: impact on clinical outcome. Nat Rev Cancer.

[86] Wherry EJ, Kurachi M (2015). Molecular and cellular insights into T cell exhaustion. Nat Rev Immunol.

[87] Austin JW, Lu P, Majumder P, Ahmed R, Boss JM (2014). STAT3, STAT4, NFATc1, and CTCF regulate PD-1 through multiple novel regulatory regions in murine T cells. J Immunol.

[88] Daud AI, Loo K, Pauli ML, Sanchez-Rodriguez R, Sandoval PM, Taravati K (2016). Tumor immune profiling predicts response to anti-PD-1 therapy in human melanoma. J Clin Invest.

[89] Thommen DS, Koelzer VH, Herzig P, Roller A, Trefny M, Dimeloe S (2018). A transcriptionally and functionally distinct PD-1^+^ CD8^+^ T cell pool with predictive potential in non-small-cell lung cancer treated with PD-1 blockade. Nat Med.

[90] Thorsson V, Gibbs DL, Brown SD, Wolf D, Bortone DS, Ou Yang TH (2018). The immune landscape of cancer. Immunity.

[91] Tumeh PC, Harview CL, Yearley JH, Shintaku IP, Taylor EJ, Robert L (2014). PD-1 blockade induces responses by inhibiting adaptive immune resistance. Nature.

[92] Mariathasan S, Turley SJ, Nickles D, Castiglioni A, Yuen K, Wang Y (2018). TGFβ attenuates tumour response to PD-L1 blockade by contributing to exclusion of T cells. Nature.

[93] Schadendorf D, Hodi FS, Robert C, Weber JS, Margolin K, Hamid O (2015). Pooled analysis of long-term survival data from Phase II and Phase III trials of ipilimumab in unresectable or metastatic melanoma. J Clin Oncol.

[94] Ribas A, Kefford R, Marshall MA, Punt CJ, Haanen JB, Marmol M (2013). Phase III randomized clinical trial comparing tremelimumab with standard-of-care chemotherapy in patients with advanced melanoma. J Clin Oncol.

[95] Sangro B, Gomez-Martin C, de la Mata M, Iñarrairaegui M, Garralda E, Barrera P (2013). A clinical trial of CTLA-4 blockade with tremelimumab in patients with hepatocellular carcinoma and chronic hepatitis C. J Hepatol.

[96] Kremer JM, Genant HK, Moreland LW, Russell AS, Emery P, Abud-Mendoza C (2006). Effects of abatacept in patients with methotrexate-resistant active rheumatoid arthritis: a randomized trial. Ann Intern Med.

[97] Reck M, Rodríguez-Abreu D, Robinson AG, Hui R, Csőszi T, Fülöp A (2016). Pembrolizumab versus chemotherapy for PD-L1-positive non-small-cell lung cancer. N Engl J Med.

[98] Kaufman HL, Russell J, Hamid O, Bhatia S, Terheyden P, D'Angelo SP (2016). Avelumab in patients with chemotherapy-refractory metastatic Merkel cell carcinoma: a multicentre, single-group, open-label, phase 2 trial. Lancet Oncol.

[99] Rittmeyer A, Barlesi F, Waterkamp D, Park K, Ciardiello F, von Pawel J (2017). Atezolizumab versus docetaxel in patients with previously treated non-small-cell lung cancer (OAK): a phase 3, open-label, multicentre randomised controlled trial. Lancet.

